# Cardiometabolic Risk Indicators That Distinguish Adults with Psychosis from the General Population, by Age and Gender

**DOI:** 10.1371/journal.pone.0082606

**Published:** 2013-12-18

**Authors:** Debra L. Foley, Andrew Mackinnon, Gerald F. Watts, Jonathan E. Shaw, Dianna J. Magliano, David J. Castle, John J. McGrath, Anna Waterreus, Vera A. Morgan, Cherrie A. Galletly

**Affiliations:** 1 Orygen Youth Health Research Centre and Centre for Youth Mental Health, University of Melbourne, VIC Australia; 2 Lipid Disorders Clinic, Metabolic Research Centre and Department of Internal Medicine, Royal Perth Hospital & School of Medicine and Pharmacology, University of Western Australia, WA Australia; 3 Department of Clinical Diabetes and Epidemiology, Baker IDI Heart and Diabetes Institute, Melbourne, VIC Australia; 4 St Vincent's Hospital, Melbourne & Department of Psychiatry, University of Melbourne, VIC Australia; 5 Queensland Brain Institute, University of Queensland & Queensland Centre for Mental Health Research, The Park Centre for Mental Health, QLD Australia; 6 School of Psychiatry and Clinical Neurosciences, University of Western Australia, WA Australia; 7 Discipline of Psychiatry, School of Medicine, University of Adelaide & Ramsay Health Care, Mental Health Services & Northern Adelaide Local Health Network, SA Australia; University College London, United Kingdom

## Abstract

Individuals with psychosis are more likely than the general community to develop obesity and to die prematurely from heart disease. Interventions to improve cardiovascular outcomes are best targeted at the earliest indicators of risk, at the age they first emerge. We investigated which cardiometabolic risk indicators distinguished those with psychosis from the general population, by age by gender, and whether obesity explained the pattern of observed differences. Data was analyzed from an epidemiologically representative sample of 1,642 Australians with psychosis aged 18–64 years and a national comparator sample of 8,866 controls aged 25–64 years from the general population. Cubic b-splines were used to compare cross sectional age trends by gender for mean waist circumference, body mass index [BMI], blood pressure, fasting blood glucose, triglycerides, LDL, HDL, and total cholesterol in our psychosis and control samples. At age 25 individuals with psychosis had a significantly higher mean BMI, waist circumference, triglycerides, glucose [women only], and diastolic blood pressure and significantly lower HDL-cholesterol than controls. With the exception of triglycerides at age 60+ in men, and glucose in women at various ages, these differences were present at every age. Differences in BMI and waist circumference between samples, although dramatic, could not explain all differences in diastolic blood pressure, HDL-cholesterol or triglycerides but did explain differences in glucose. Psychosis has the hallmarks of insulin resistance by at least age 25. The entire syndrome, not just weight, should be a focus of intervention to reduce mortality from cardiovascular disease.

## Introduction

All-cause mortality in individuals with schizophrenia is more than twice that of the general population [1]. Life expectancy is reduced by 10–25 years [2]. A common cause of that premature death is coronary heart disease [3]. Interventions to improve cardiovascular health should be implemented as early as possible but the age when many potentially modifiable indicators of risk first diverge in those with psychosis relative to the general population is unknown. Weight gain is an early and common side effect of antipsychotic drug treatment [4] and elevated body weight and waist circumference are risk factors for insulin resistance [5] and coronary heart disease [6]. How much of the broader cardiometabolic risk profile associated with psychosis across adulthood is driven by weight or central obesity is unknown. Type 2 diabetes and heart disease both reflect the cumulative effect of multiple risk factors. Typically, these risk factors have a threshold above which they are considered clinically significant. However, multiple elevated but sub-threshold risk factors can also be clinically significant and could be used to identify an emerging risk factor profile. Left untreated that profile may hasten morbidity and mortality but if identified early could be an effective target for intervention.

The aims of this study were to determine 1] the age key cardiometabolic risk indicators first diverge from the general population in those with psychosis, 2] the pattern of cardiometabolic risk indicators that characterizes psychosis across adulthood, and 3] the extent to which elevated body mass index and waist circumference explains the observed pattern of cardiometabolic risk indicators associated with psychosis during adulthood. We achieved aims 2] and 3] but aim 1] could not be determined because the distinctive cardiometabolic profile that distinguished psychosis was already present at age 25.

## Materials and Methods

### Ethics statement

The research protocol for the psychosis study was approved by the University of Western Australia Human Research Ethics Committee, the Human Research Ethics Committee at St Vincent's Health Melbourne, the Central Northern Adelaide Health Services Ethics of Human Research Committee, the West Moreton Health Service District Human Research Ethics Committee, the Melbourne Health Human Research Ethics Committee, the South Metropolitan Area Health Service Human Research Ethics Committee, the Greater Western Area Health Service Human Research Ethics Committee, the University of Newcastle Human Research Ethics Committee and the Hunter New England Research Ethics Committee.

Written informed consent was obtained from subjects after complete description of the study. Individuals with psychosis strongly endorse the importance of autonomous decision-making [7] and psychotic symptoms (e.g., hallucinations, delusions) are not significantly associated with diminished decisional capacity [8]. The survey excluded those with insufficient English or a communication or cognitive impairment that would interfere with a person's capacity to give informed consent and to complete a valid interview and those unavailable for screening or interview due to residence in a nursing home or prison. Several safeguards were also put in place to ensure that subjects were consented appropriately. Interviewers, who judged the subject's capacity to consent, were all experienced mental health professionals. Information about the study was presented in an enhanced visual format to limit difficulties in comprehending or retaining information and consent to participate was sought only at a time when interviewers were confident that the subject's psychosis did not interfere with their capacity to give that consent. Anyone deemed unable to provide informed consent was excluded from the survey.

The research protocol for the general population survey was approved by the Ethics Committee of the International Diabetes Institute. Written informed consent was obtained after complete description of the study to the subjects.

### Samples

The psychosis sample is described in detail elsewhere [9]. In brief, the Study of High Impact Psychosis [SHIP] is a population-based cross-sectional study of psychosis. The target population was defined by diagnosis, place of residence, age 18–64 years and contact with public mental health services or relevant non-government organizations. The study was conducted in 2010 at seven sites across the five mainland Australian states. Sample weights were devised based on age and site and were derived from a census of those in contact with services at each site. Data analyzed here are for the 1,642 participants aged 18–64 with a diagnosis of ICD-10 [10] psychosis who also had cardiometabolic data, n = 1,052 to 1,598. Psychosis diagnoses were schizophrenia [53%, n = 857], bipolar disorder, mania [20%, n = 319], schizoaffective disorder [16%, n = 293], depressive psychosis [5%, n = 81] and delusional disorders or other non-organic psychoses [6%, n = 92].

The general population sample is described in detail elsewhere [11]. In brief, the Australian Diabetes, Obesity and Lifestyle study [AusDiab] is a national population-based longitudinal study of diabetes and cardiometabolic risk factors. The target population was defined by place of residence and age 25+. The baseline survey for AusDiab was conducted in 1999/2000 at six clusters within each of the six states and the Northern Territory of Australia. Sample weights were devised based on response rates and coverage of the population within the sampling frame to obtain nationally representative estimates. Data analyzed here are for the 8,866 participants aged 25–64 years who completed the baseline survey who also had cardiometabolic data, n = 8567 to 8864, and hereafter referred to as control data.

### Measures

The Diagnostic Interview for Psychosis [12] was used to diagnose psychosis and psychosis subtypes in SHIP. The physical health assessment included measurement of waist circumference, BMI, systolic and diastolic blood pressure, fasting plasma glucose, total, LDL and HDL cholesterol and triglycerides in SHIP and AusDiab [13], [14]; all have thresholds to define at-risk status in the general population [Table 1] and all metabolic assays were carried out using standard enzymatic methods that were quality assured. Drug treatment for psychosis, hypertension, elevated cholesterol, hyperglycemia or diabetes was recorded based on self-report in AusDiab and medication chart review in SHIP.

**Table 1:** Measures of obesity, dyslipidemia, diabetes and hypertension and associated risk thresholds.

			Male Prevalence (95% CI) Raw N		Female Prevalence (95% CI) Raw N	
Domain	Measure	Risk Threshold	General population sample	Psychosis sample	General population sample	Psychosis sample
**Obesity**	**BMI**	**>25 (Overweight)**	0·48 (0·46–0·51) 2468/4995	0·33 (0·30–0·36) 325/991	0·30 (0·28–0·32) 1979/6072	0·25 (0·21–0·28) 147/607
	**BMI**	**>30 (Obese)**	0·19 (0·18–0·21) 1044/4995	0·45 (0·41–0·48) 430/991	0·22 (0·20–0·24) 1434/6072	0·55 (0·51–0·59) 327/607
	**Waist**	**94 cm (M) 80 cm (F)**	0·55 (0·53–0·57) 3046/4987	0·79 (0·76–0·81) 758/986	0·57 (0·55–0·59) 3744/6081	0·93 (0·91––0·95) 554/603
**Hypertension**	**Systolic BP**	**130 mmHg**	0·53 (0·51–0·55) 2874/5020	0·35 (0·31–0·38) 341/987	0·38 (0·36–0·40) 2528/6171	0·21 (0·17–0·24) 127/603
	**Diastolic BP**	**85 mmHg**	0·21 (0·19–0·23) 1094/5021	0·43 (0·40–0·47) 420/987	0·10 (0·09–0·12) 529/6171	0·43 (0·39–0·48) 252/603
**Dyslipidemia**	**Total-c^1^**	**5·6 mmol/L**	0·47 (0·45–0·50) 2595/5047	0·30 (0·26–0·33) 217/711	0·48 (0·46–0·50) 3224/6198	0·31 (0·26–0·36) 137/444
	**LDL-c^1^**	**2·0 mmol/L**	0·97 (0·96–0·98) 4694/4804	0·88 (0·85–0·90) 556/634	0·95 (0·94–0·96) 5873/6089	0·86 (0·82–0·89) 361/418
	**HDL-c^1^**	**1·03 mmol/L (M) 1·29 mmol/L (F)**	0·21 (0·19–0·23) 1112/5042	0·49 (0·45–0·53) 348/705	0·23 (0·22–0·25) 1532/6197	0·52 (0·47–0·57) 233/440
	**Triglycerides^1^**	**1·70 mmol/L**	0·34 (0·32–0·36) 1917/5047	0·52 (0·48–0·56) 370/710	0·24 (0·23–0·26) 1662/6198	0·46 (0·41–0·51) 200/444
**Diabetes**	**Glucose^1^**	**5·6 mmol/L**	0·44 (0·42–0·46) 2476/5047	0·32 (0·29–0·36) 218/709	0·25 (0·23–0·27) 1702/6198	0·27 (0·23–0·32) 114/443

Key for Table 1:

M = Male; F = Female; 1 = estimated from fasting blood sample; All differences between the psychosis and the general population samples were significant (p<·001) except overweight BMI in females (p<·01) and fasting blood glucose in females (p = 0·39).

### Statistical Analysis

Data analysis was undertaken using Stata v12.1. All parameter estimation used sampling weights from each sample. The prevalence of drug treatments between samples was compared using a z-test. Pearson's correlation coefficient was used to estimate associations between variables. Cubic b-splines [15] for age in years were generated using the Stata bsplines procedure and fitted to each of the cardiometabolic risk indicators, separately by sex by sample, yielding smoothed means across the age span. These curves provide a relative unconstrained picture of differences in each indicator over the lifespan but smooth minor variations due to sampling. Substantial non-overlap of the confidence regions [plotted as ±1se] for the smoothed mean for each curve provides an informal indication of the significant differences between the psychosis and control samples for risk indicators at particular ages or age ranges. The at-risk threshold for each indicator is included on each plot. The age the average value for the psychosis sample first exceeded each threshold was estimated from the fitted models. We note when the estimated age was outside the range of observed values and infer on that basis that the threshold was exceeded before age 18. Age splines for blood pressure, lipids and glucose for the psychosis sample were also fitted controlling for BMI and waist circumference and estimated at the age and sex specific means of these variables in controls to test if observed sample differences were due to differences in mean BMI and central obesity.

The means for all risk indicators were compared within five age bands (18–24; 25–34; 35–44; 45–54; 55–65) in those with affective versus non-affective psychoses to test for potential heterogeneity in our findings. Affective psychosis was defined as schizoaffective, bipolar, mania, depressive psychosis and non-affective psychosis was defined as schizophrenia, delusional & other non-organic psychosis. These analyses were conducted separately for males and females. Adjustment for multiple comparisons used the relatively efficient Holm-Bonferroni method [16].

## Results

### Comparative pattern of cardiometabolic risk indicators

**Figure 1:**
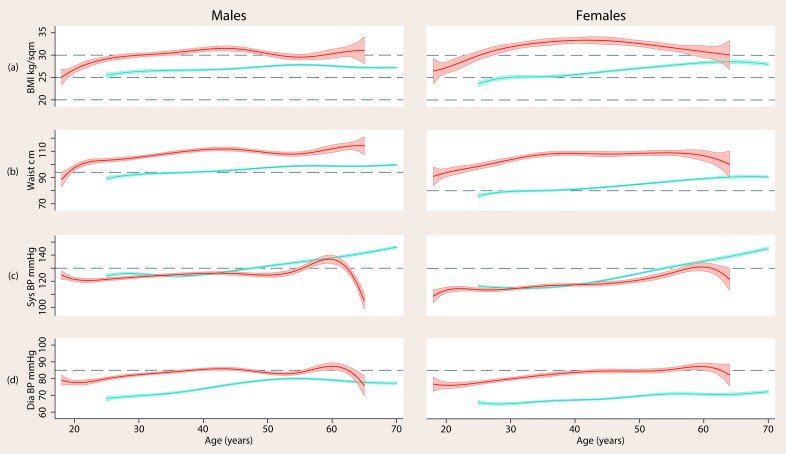
Smoothed means of body mass index [BMI], waist circumference, systolic and diastolic blood pressure by age in men and women with psychosis [red] compared to general population controls [blue]. Shaded areas indicate ±1 standard error of the mean. Dashed lines indicate commonly used community thresholds of risk status [see Table 1].

**Figure 2:**
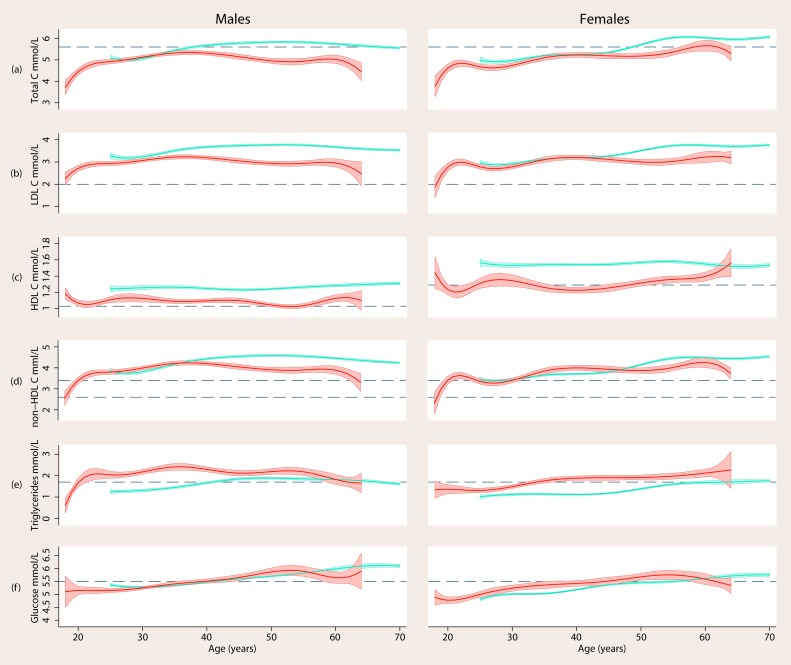
Smoothed means of fasting blood total, LDL and HDL cholesterol, triglycerides and glucose by age in men and women with psychosis [red] compared to general population controls [blue]. Shaded areas indicate ±1 standard error of the mean. Dashed lines indicate commonly used community thresholds of risk status [see Table 1].

The same pattern of risk indicators distinguished psychosis at all ages [Figures 1 and 2]: from at least age 25 individuals with psychosis had significantly higher mean BMI, waist circumference, diastolic blood pressure, triglycerides and glucose [women only] and significantly lower HDL-c than controls [Table 2]. These differences were observed at all ages, with the exception of triglycerides at age 60+ in men [Figure 2d] and glucose which at some ages was significantly worse in women with psychosis and at some ages not [Figure 2e]. Systolic blood pressure did not differ between samples at any age and, for men, neither did glucose. Men with psychosis had significantly lower LDL-c from age 25 and significantly lower total cholesterol from age 35 than controls. Women with psychosis had significantly lower LDL-c from age 46 and significantly lower total cholesterol before age 33 and after age 45 than controls. Non-HDL-c [total–HDL-c] may be elevated when LDL-c is not but that was not the case here. The correlation between non-HDL-c and total cholesterol in both samples was >0.9.

**Table 2:** Age at which the mean for measures of obesity, dyslipidemia, diabetes and hypertension first exceeded standard risk thresholds.

		Male			Female		
Domain	Risk Indicator (Mean)	Rank (Psychosis)	Age		Rank (Psychosis)	Age	
			Psychosis	Controls		Psychosis	Controls
Dyslipidemia	Cholesterol-LDL	1	-∧	-∧	1^ = ^	-∧	-∧
Obesity	BMI overweight	2	18·0*	23·4†	2	18·2	29·3
Obesity	Waist circumference	3	19·1	38·0	1^ = ^	-∧	34·1
Dyslipidemia	Triglycerides	4	20·1	41·2	4	33·7	62·3
Obesity	BMI obese	5	29·1	-v	5	25·1	-v
Dyslipidemia	Cholesterol-HDL	6	29·3	-∧	3	24·4‡	-∧
Diabetes	Glucose	7	43·5	45·0	6	47·7	59·4
Hypertension	Diastolic BP	8	46·5	-v	7	53·5	-v
Hypertension	Systolic BP	9	55·4	47·5	8	57·7	53·8
Dyslipidemia	Cholesterol-total	10	-v	37·9	9	58·3	48·7

Key to Table 2:

† Estimate outside the age period of observation.

v Always below threshold within the period of observation.

∧ Always above threshold within the period of observation.

‡ HDL cholesterol is transiently above threshold at the extreme of the age range of observations (18–19·2).

*Occurs right at the edge of the age period of observation.

### Impact of adjustment for BMI and waist circumference

In those with psychosis the correlations of BMI and waist circumference with other risk indicators ranged from 0.01 to 0.33 [0.18 to 0.42 in controls]. Adjusting risk indicator model estimates in the psychosis sample to the mean BMI and waist circumference of controls explained all of the observed elevation in glucose in women with psychosis and drove other indicators that differed significantly between samples towards the values observed in controls but did not explain all the differences. For example, at the mean age of the psychosis sample, 38.6 years for men and 40.7 years for women, adjustment for BMI and waist circumference explained 22% of the difference in diastolic blood pressure in men and 24% in women, 26% of the difference in HDL-c in men and 29% in women, and 32% of the difference in triglycerides in men and 60% in women.

### Age at which means exceeded the risk factor thresholds in psychosis sample

The psychosis sample mean exceeded at-risk thresholds for LDL-c at or below age 18, body mass and central obesity at or below age 18–19, triglycerides at age 20 in women and age 34 in men, HDL-c at age 25 in men and age 29 in women, fasting plasma glucose at age 43 in women and age 48 in men, diastolic hypertension at age 46 in women and age 53 in men and systolic hypertension at age 55 in women and age 58 in men [Table 3].

**Table 3:** The age or age range when cardiometabolic risk indicators were significantly different in those with psychosis compared with general population controls.

Domain	Risk Indicator	Males with psychosis 25–64 years	Females with psychosis 25–64 years
**Obesity**	**Waist circumference**	Always worse	Always worse
	**BMI**	Always worse	Always worse
**Dyslipidemia**	**Cholesterol-Total**	Better after age 35^1^	Better after age 45^2^
	**Cholesterol-LDL**	Always better^3^	Comparable to ∼ age 40^4^
	**Cholesterol-HDL**	Always worse	Always worse
	**Triglycerides**	Worse until ∼60^5^	Always worse
**Hypertension**	**Systolic BP**	Generally slightly better^6^	Generally slightly better^7^
	**Diastolic BP**	Always worse	Always worse
**Diabetes**	**Glucose**	Generally comparable^8^	Generally comparable^9^

Key to Table 3:

1 Mean is lower after age 33 but CIs do not overlap after age 35.

2 Lower at all ages although between age 38 and 42 the difference is less than ·05 - lower than the accuracy of the assay. Before 33 and after 45 CIs do not overlap.

3 CIs never actually coincide, but from 27–31 the gap between the lower bound of the general population sample and the upper bound of the psychosis sample is 0·0 to one decimal place.

4 After age 46 the CIs do not overlap. The psychosis sample mean is lower from age 40.

5 Means for triglycerides in males from the psychosis and general population samples meet at 60. CIs overlap from age 57.

6 Men from the general population sample have higher mean systolic blood pressure only between age 34 and 41. CIs always overlap, so the difference is not significant.

7 Lower mean systolic blood pressure only between age 32 and 40 but CIs always overlap.

8 Blood glucose means are higher from age 31 to 56 although CIs overlap during this period.

9 Blood glucose means are higher up to age 60. CIs sometimes overlap, sometimes don't during this period.

### Treatment for elevated cholesterol, hypertension or diabetes

Between age 25–64 individuals with psychosis were 5.7 times more likely to be treated for diabetes [9.1% versus 1.6%, z = 8.84 p<0.0001], 1.65 times more likely to be treated for hypertension [12.26% versus 7.41%, z = 4.72 p<0.0001] and 2.8 times more likely to be treated for elevated cholesterol [13.21% versus 4.72%, z = 8.37 p<0.0001] than controls.

### Antipsychotic drug treatment

At the time of the psychosis survey 85% of the 1,642 participants had been prescribed antipsychotic medication in the previous four weeks: 64% received second generation [atypical] antipsychotic medication, 11% first generation [typical] antipsychotic medication and 10.0% had been prescribed both. The most commonly prescribed antipsychotic drugs were all atypicals: risperidone 25%, quetiapine 25%, clozapine 21% and olanzapine 18%.

### Tests for heterogeneity

Thirteen of the 90 comparisons undertaken in those with affective versus non-affective psychosis were statistically significant (p<.05) but only one (blood glucose in males aged 18–24) survived within variable adjustment for multiple comparisons using the relatively efficient Holm-Bonferroni method. Significant unadjusted differences tended to be sporadic, occurring in only isolated age groups inconsistently for males and females.

## Discussion

### Comparative pattern of cardiometabolic risk indicators

Insulin resistance is characterized by obesity, hypertension, hyper-triglyceridemia, lowered HDL-c, sometimes elevated LDL-c but more often elevated non-HDL-c, and ultimately in some cases hyperglycemia and type 2 diabetes [17]. The pattern of cardiometabolic risk indicators associated with psychosis here may therefore reflect a propensity to develop insulin resistance. Cross-sectional age trends for cardiometabolic risk indicators in psychosis, by gender, have not previously been published. Once seen, however, it is clear that individuals with psychosis are very different from the general population from a very early age, at least age 25, but how early for every indicator we cannot say. Studies designed to estimate the national incidence of type 2 diabetes and characterize associated cardiometabolic risk factors, like the AusDiab study that served as our control sample, do not typically ascertain subjects prior to early adulthood.

Insulin resistance is a precursor of type 2 diabetes, it underlies the metabolic syndrome and is a risk factor for coronary heart disease [17]. Insulin resistance may therefore be a common pathway for the elevated risk for all three conditions in those with psychosis, and an important cause of their elevated cardiovascular morbidity. Around half of all deaths in those with type 2 diabetes are due to cardiovascular disease [18].

### Impact of adjustment for BMI and waist circumference

Obesity is a common driver of insulin resistance [5]. The mean body weight of the psychosis sample exceeded the overweight BMI threshold at age 18. The mean waist circumference of the psychosis sample exceeded the risk threshold before age 18 in women and at age 19 in men. Nonetheless, BMI and waist circumference could not explain all the observed differences in diastolic blood pressure, HDL-c or triglycerides between samples. Some of the weight differences between samples may be caused by insulin resistance. Insulin resistance will increase appetite and blunt satiety signals; excess circulating glucose will be converted to fat by insulin. Insulin resistance in the heart could also have a direct negative effect on heart function. Intervention to improve the physical health of individuals with psychosis should therefore target the entire insulin resistance syndrome, not just weight [19].

### Earliest age at which risk factor thresholds were exceeded in psychosis sample

This could not be estimated for LDL-c because the sample mean for those with psychosis already exceeded the at-risk threshold at age 18. In contrast to those with psychosis, mean BMI, HDL-c and diastolic blood pressure readings for those in the general population never exceeded the thresholds that define obesity, low HDL-c or hypertension.

### Antipsychotic drug treatment effects

Psychosis onset occurred before age 25 in two-thirds of our psychosis sample [9]. Duration of psychosis is therefore correlated with age. Most individuals with psychosis were currently taking antipsychotic drugs and their length of use is correlated with duration of psychosis and therefore with age. If duration of illness/antipsychotic drug treatment explained the cross-sectional age trends reported here, risk would get worse with age *relative to controls*. That's not what we observed; differences actually diminished a little with age. Controls get worse with age but those with psychosis are also more likely to die with increasing age. Any effect of duration of illness/treatment on emerging insulin resistance must occur relatively early in the course of illness or treatment, and in our psychosis sample by age 25. Experimental studies of the very short-term [3 day] effects of olanzapine on healthy controls have reported a pattern of findings very similar to our own: lowered glucose tolerance, lowered HDL-c, elevated triglycerides but not LDL-c or total cholesterol [20]. A review of early cardiometabolic outcomes of treated psychosis described a significant effect of antipsychotic drugs on BMI and waist circumference, triglycerides, HDL-c and glucose and, unlike here, LDL-c and total cholesterol [4]. However, those results are a compilation of findings across multiple studies and many focused only on weight change. The largest most comprehensive studies, such as the EUFEST trial [21], did not discuss the changing pattern of cardiometabolic indicators after initiation of antipsychotic drug treatment but focused instead on differences in 1 year outcomes for patients randomized to different drugs. Treatment effects data beyond 1 year are so sparse due to the extremely high rate of participant withdrawal that they cannot be considered representative [6]. It is only recently that the annual incidence of diagnosed diabetes in those treated with antipsychotic drugs has been estimated using large population-based data [22].

### Heterogeneity

The study did not identify any consistent differences in cardiometabolic risk indicators between participants diagnosed with an affective versus a non-affective psychosis. In some instances this may reflect lack of power as the number of participants was quite modest at the extremes of the age distribution for some age bands. Elucidation of differences between individual psychosis diagnoses is likely to require larger studies given the overall pattern of age and sex differences observed here.

Antipsychotic drugs are not the only reason for the observed propensity to insulin resistance in those with psychosis. An association between diabetes and schizophrenia has been noted for over a century and long before the advent of antipsychotic drugs [23], [24]. This is consistent with a higher prevalence of abnormal glucose tolerance and increased visceral fat, both signs of insulin resistance, in antipsychotic drug naïve cases compared with controls [25], [26]. Some consider abnormal glucose tolerance a risk equivalent to type 2 diabetes for coronary heart disease [27]. Risk for type 2 diabetes is then further increased by exposure to antipsychotic drugs [28]. This varies by drug, with the greatest risk observed in association with low potency first generation antipsychotic drugs and with two second generation drugs, olanzapine and clozapine [22]. This has led to recommendations that certain drugs should not be first line treatment options [24]. Others have suggested low drug doses [29], [30], extended [non-daily] dosing [31], [32] or drug switching [33] to limit adverse effects.

Change in cardiometabolic risk indicators after commencement of any antipsychotic drug should be closely monitored. Monitoring guidelines have been developed and evaluated [34] but they have had little impact on clinical practice [35]. This is an important failure of care by those who prescribe antipsychotic drugs. Mandated monitoring systems have been proposed [36] but this alone won't change the “scandal of premature mortality” associated with psychosis [37]. It is simply a first, necessary, step. Effective ongoing medical management of treated psychosis is then required [38].

### Caveats and limitations

The lives and lifestyles of those with psychosis differ in many ways from those in the general population. It therefore should not be assumed that all differences in cardiometabolic risk indicators can be attributed to either antipsychotic drugs or the unmeasured risk factors that underlie the expression of psychosis, although both play a role. Individuals with psychosis are more likely to smoke cigarettes, have a poor diet and be physically inactive [39]. The same caveats apply to these potential modifiers of risk as to the effects of psychosis and antipsychotic drug treatment; they must exert their effects early to generate the pattern of risk indicators observed in the present study at age 25. Our results reflect the current status of surveyed individuals given any past or current drug treatment, irrespective of efficacy. Medication for psychosis, hypertension, dyslipidemia or hyperglycemia could alter values on cardiometabolic risk indicators, and may partly explain the unexpectedly lower mean level of LDL, non-HDL and total cholesterol in those with psychosis at some ages. The pattern of dyslipidemia associated with psychosis here therefore requires further investigation in other samples, including assessment of ApoB, remnant like particles and small dense LDL-c which SHIP did not measure. However, psychosis itself may also be associated with an abnormal lipid profile [40].The present study was conducted in one developed country [Australia] and the pattern of observed risk given any current drug treatment will therefore partly reflect national prescribing patterns and other factors that determine access to health care. Replication is now needed in other data sets from other countries to determine the degree of international variation in observed patterns of multiple cardiometabolic risk indicators by age in association with psychosis.
